# Modulation of TcR/CD3-zeta chain expression by a circulating factor derived from ovarian cancer patients

**DOI:** 10.1054/bjoc.2001.1847

**Published:** 2001-06

**Authors:** D D Taylor, D P Bender, Ç Gerçel-Taylor, J Stanson, T L Whiteside

**Affiliations:** Departments of Obstetrics & Gynecology and Radiation Oncology, University of Louisville School of Medicine, Louisville, KY, 40292; University of Pittsburgh Cancer Institute, Pittsburgh, PA 15213, USA

**Keywords:** TcR/CD3-zeta, immunosuppression, ovarian cancer

## Abstract

In women with ovarian cancer, suppression of components of the immune system may promote tumour development. Previous studies in ovarian cancer have demonstrated that decreased expression and function of the T-cell receptor (TcR)-associated signal transducing zeta-chain correlates with deficient immune responsiveness of T cells. In this study, sera and ascitic fluids obtained from woman with advanced ovarian cancer were found to suppress the expression of TcR-associated zeta chain. This suppression of zeta chain expression was dose-dependent and was not observed with biologic fluids obtained from healthy women. © 2001 Cancer Research Campaign http://www.bjcancer.com


*British Journal of Cancer* (2001) **84**(12), 1624–1629
© 2001 Cancer Research Campaign
doi: 10.1054/ bjoc.2001.1847, available online at http://www.idealibrary.com on IDEAL®

### Modulation of TcR/CD3-zeta chain expression by a circulating factor derived from ovarian cancer patients

**DD Taylor[1], DP Bender[1], Ç Gerçel-Taylor[1], J Stanson[2] and TL Whiteside[2]**

[1]Departments of Obstetrics & Gynecology and Radiation Oncology, University of Louisville School of Medicine, Louisville, KY, 40292; [2]University of Pittsburgh Cancer Institute, Pittsburgh, PA 15213, USA

**Summary** In women with ovarian cancer, suppression of components of the immune system may promote tumour development. Previous studies in ovarian cancer have demonstrated that decreased expression and function of the T-cell receptor (TcR)-associated signal transducing zeta-chain correlates with deficient immune responsiveness of T cells. In this study, sera and ascitic fluids obtained from woman with advanced ovarian cancer were found to suppress the expression of TcR-associated zeta chain. This suppression of zeta chain expression was dose-dependent and was not observed with biologic fluids obtained from healthy women.

The factor responsible for the loss of zeta chain was purified from ascites and characterized as a protein with an appropriate molecular weight of 14 kD. Suppression of T-cell TcR-zeta was specific, since neither *lck* nor ZAP-70 expression was affected, while zeta chain was almost completely suppressed. This selective suppression of TcR-zeta expression by the 14 kD ascites-derived factor was shown to operate at the mRNA level. By defining the mechanism through which this protein modulates TcR-zeta chain levels, it might be possible to ultimately prevent the suppressive influences of the tumour microenvironment and restor immune competence in patients with ovarian carcinoma. © 2001 Cancer Research Campaign http://www.bjcancer.com

**Keywords**: TcR/CD3-zeta; immunosuppression; ovarian cancer

Suppression of immune cell functions is one of the manifestations of progressive cancer and is a critical factor in the prognosis of cancer patients (Old, 1981; Hellstrom and Hellstrom, 1991). The impairments observed in lymphocytes are often subtle as reflected by variably decreased, but not absent, immune functions and are more pronounced at the tumour site than in the peripheral circulation of patients with cancer. Evidence indicates that the tumour itself exerts these immunosuppressive effects, including decreased monocyte/macrophage functions (Bast and Knapp, 1984) and diminished cellular immunity as manifested by decreased numbers of circulating lymphocytes, their reduced responsiveness to mitogens and by decreased delayed cutaneous hypersensitivity (DTH) observed in patients with high tumour burdens (Braun and Harris, 1983; Brooks and Rees, 1988).

Functional abnormalities in T lymphocytes have been observed in patients with ovarian, cervical, renal cell, colorectal, prostate and head and neck cancers, including defective $Ca^{2+}$ mobilization, impaired tyrosine kinase activity, and decreased expression of TcR-associated zeta and epsilon chains (Tartour et al, 1995; Lai et al, 1996; Rabinowich et al, 1996; Mulder et al, 1997; deGruijl et al, 1999; Kuss et al, 1999). In patients with gynaecologic malignancies, the diminished expression of the signal-transducing zeta chain has been observed in both tumour-infiltrating T cells and peripheral blood lymphocytes of patients with advanced - cancer (Lai et al, 1996; Rabinowich et al, 1996; deGruijl et al, 1999). Decreased expression of CD3-zeta has been correlated with reduced proliferative responses following antigenic challenge and reduced cytokine production. Other investigators have correlated the decreased expression of zeta chain in cancer patients' T-lymphocytes with extent of disease and short survival (Shores et al, 1997).

*Received 8 November 2000*
*Revised 15 March 2001*
*Accepted 20 March 2001*

*Correspondence to:* DD Taylor

Investigations into the molecular mechanisms leading to the reduced zeta expression in advanced ovarian cancer have indicated that it appeared to result from increased degradation and not from decreased synthesis (Reichert et al, 1998). In vitro studies of the mechanism of decreased zeta chain expression have demonstrated its level could be diminished by direct tumour cell-lymphocyte contact (Rabinowich et al, 1998). However, evidence for the systemic inhibition of T-cell zeta chain expression has led some investigators to suggest the presence of a circulating suppressive factor (Yasumura et al, 1994). The nature of this systemic inhibition and the role of a putative circulating factor remain undefined.

While the loss of zeta chain is a common observation, the mechanism responsible for the cancer-associated decreased expression of T-cell zeta chain remains controversial. This study reports the identification of a circulating factor isolated from ovarian cancer patients that can mediate suppression of CD3-zeta chains. Defining the mechanism, through which this factor modulates zeta chain levels, might ultimately provide a target for the prevention of the suppressive influences of the tumour microenvironment, leading to the generation of effective anti-tumor cellular immune responses and restoration of protective immune responses in patients with cancer.

### MATERIALS AND METHODS

### Patient materials

Blood specimens and ascitic fluids were obtained from ovarian cancer patients ($n$ = 30) in the Gynecologic Oncology Clinic of the Department of Obstetrics and Gynecology of the University of Louisville School of Medicine. The specimens were collected as part of an informed consent protocol approved by the University Human Studies Committee. Blood samples were also obtained from age-matched normal female volunteers ($n$ = 15) recruited from patients in the Gynecology Clinic of the Department of Obstetrics and Gynecology of the University of Louisville School of Medicine. Blood samples were allowed to clot and then were centrifuged at 400 ***g*** for 10 minutes to sediment cells and clot. The serum was removed, aliquoted, and stored at –70˚C until analysis.

Ascitic fluids were also obtained from women with ovarian cancer ($n$ = 15). Ascitic fluids were initially treated with heparin, cells and cellular debris were removed by centrifugation and the fluid was filtered sterile using a 0.45 µm filter. These ascites samples were aliquoted and stored at –70˚C until analysis.

### Jurkat cells and culture conditions

To analyse T-cell modulation and activation, Jurkat cells, (Jurkat E6-1 cells) a human T-cell lymphoma, were used. These lymphoma cells were obtained from the American Type Culture Collection (Rockville, MD). They were selected for this study because they possess a functional TcR/CD3 complex and can be induced to synthesize and secrete interleukin-2 (IL-2). Jurkat cells were routinely grown in RPMI 1640 medium supplemented with 10% fetal bovine serum, 0.1 mM nonessential amino acids, 1 mM sodium pyruvate, 200 mM L-glutamine, 100 mg $ml^{-1}$ streptomycin and 100 IU $ml^{-1}$ penicillin in a humidified 5% $CO_2$ atmosphere. They were harvested in the log phase of growth and their viability was evaluated by a trypan blue dye exclusion. All cultures utilized for this study were >95% viable.

### Western immunoblot assay for lymphocyte protein expression

To assess the effects induced by sera or ascitic fluids obtained from ovarian cancer patients, Jurkat cells ($10^6$ cells $ml^{-1}$) were incubated in medium supplemented with 0 to 25% patient-derived fluids for 4 days. After 4 days, the cells were centrifuged, the cell pellets washed and subsequently lysed using 50 mM HEPES, pH 7.2, 150 mM sodium chloride, 5 mM EDTA, 1 mM sodium-orthovanadate, 2.5% Triton X-100, 200 µg $ml^{-1}$ trypsin/chymotrypsin inhibitor, 200 µg $ml^{-1}$ chymostatin and 2 mM PMSF. The cell lysate was assayed for protein by the Bradford Coomassie brilliant blue assay (Bio-Rad Laboratories, Richmond, CA) and 30 µg of the protein from each cell lysate was applied per lane to a 15% SDS-PAGE gel. The proteins were electrophoretically separated by the method of Laemmli (1970) and transferred to nitrocellulose paper for analysis by Western immunoblot (Brown et al, 1993). The blots were blocked with 10% nonfat dry milk and probed overnight at 4˚C in the same buffer with either mouse anti-CD3-zeta, anti-*lck*, or anti-ZAP-70 monoclonal antibodies (Santa Cruz Biotechnology, Santa Cruz, CA) as the primary antibodies. After this incubation, the membranes were washed 3 times in 0.1% Tween 20 in TBS for 15 minutes. The blots were then incubated in blocking buffer with rabbit anti-mouse IgG with horseradish peroxidase (Sigma Chemical Co, St. Louis, MO) for 45 minutes at room temperature. The immunoblots were washed 3 times in 0.1% Tween 20 in PBS, after which bound complexes were visualized by enhanced chemiluminescence (ECL, Amersham Life Sciences, Arlington Heights, IL).

To define the specificity of decreased zeta chain expression, partially purified zeta suppressor factors (10 ng $ml^{-1}$) were prepared from the ascites of 5 patients and also from the analogous fraction from a control serum. Jurkat cells ($5 \times 10^6$ cells) were incubated with these fractions for 96 hours, the cells were isolated, and cellular protein expression was examined by Western immunoblot for the levels of zeta chain as well as of *lck* and ZAP-70 as described above.

### Confirmation of CD3-zeta suppression in normal lymphocytes

Blood specimens from healthy donors were also collected to isolate normal T-lymphocytes. The blood was collected in sterile tubes with the addition of Heparin (1000 U $L^{-1}$) and peripheral blood mononuclear cells (PMNC) were obtained by density gradient centrifugation using Ficol-Hypaque. Further, monocytes/macrophages were depleted through plastic adherence for 45 minutes at 37˚C to obtain peripheral blood lymphocytes (PBL). PBL ($5 \times 10^6$ cells) from 5 normal volunteers were incubated with the partially purified zeta suppressor factor (10 ng $ml^{-1}$) for 96 hours. Purified T cells were isolated by magnetic selection, as previously described (Lai et al, 1996). The resulting T-cells, which were >98% CD3+ cells, were isolated and lysed. The level of CD3-zeta expression was examined by Western immunoblot, as described above.

### Purification of the zeta suppressive factor

After the initial studies demonstrated zeta chain suppression in Jurkat cells incubated in the presence of patients' sera and ascitic fluids, further studies were initiated to purify the factor(s) responsible for this effect. The factor inducing suppression of CD3-zeta was partially purified from 5 patient ascitic fluids known to contain this activity. Initially, ascitic fluids (250 ml each) were centrifuged at 400 ***g*** for 10 minutes to remove cells and then filtered through a 500 kD molecular weight (mw) ultrafiltration membrane. These ascitic fluids were incubated with Affi-Gel Blue (Bio-Rad Laboratories, Richmond, CA) to remove albumin and concentrated using a 10 kD mw cutoff ultrafiltration membrane. This material was then fractionated on the basis of molecular weight by molecular weight using a Vydac GPC60/150 column (4.6 × 500 mm). The column was run isocratically, using PBS as the mobile phase. The column was run at 200 µl $min^{-1}$, monitoring the elution at 214 nm. The resulting fractions were assayed for the zeta suppressing activity and the fractions containing this activity were pooled and further fractionated by reverse phase-HPLC on a Vydac C8 (300 µl) RP-HPLC column (4.6 × 250 mm). The column was eluted at a flow rate of 1.0 ml $min^{-1}$ with a linear gradient of acetonitrile: water: trifluoroacetic acid (0:99.9:0.1 at time zero to 99.9:0:0.1 at 60 minutes) and 1 minute fractions were collected. An aliquot of each fraction was assayed for suppression of zeta chain expression in Jurkat cells. The positive material was pooled and analysed using MALDI-TOF on a sinapinic acid matrix by Commonwealth Biotechnologies (Richmond, VA).

© 2001 Cancer Research Campaign

### Identification of ZIP target within the T-cell

To identify the pathway leading to reduced expression of zeta chain as defined by Western immunoblot, expression of mRNA for zeta chain was examined in Jurkat cells co-incubated with the factor, as previous described (12). Reverse transcription (RT) polymerase chain reaction (PCR) was performed under conditions previously described (Suminami et al, 1995). Jurkat cells ($10^6$ cells $ml^{-1}$) were incubated with partially purified zeta suppressor factor (10 ng $ml^{-1}$) or the analogous fraction from a control serum for 4 days. Total cellular RNA was then extracted from $10^6$ Jurkat cells. A hot PCR with [$\alpha$-$^{32}$P]dCTP was performed using cDNA obtained by RT of 100 ng of cellular RNA using sense/antisense primers: for CD3-zeta chain protein, GTTGCCGATTACAGAG-GCAC and TTGGTGGCTGTACTAAGACC and for β-action, as a control, GGGTCAGAAGGATTCCTATG and GGTCTCAAA-CATGATCTGGG. The amplification was performed by denaturing at 94˚C for 60 seconds, annealing at 59˚C for 60 seconds, and DNA chain extension at 72˚C for 60 seconds. A total of 25 cycles was used for amplification. The amplified products were then separated on 5% polyacrylamide gels and the radioactive signals were quantitated using a PhosphorImager SI. The ratio of the signal for the zeta chain to that for β-action was calculated and the zeta/action ratios obtained for treated Jurkat cells were compared with untreated Jurkat cells.

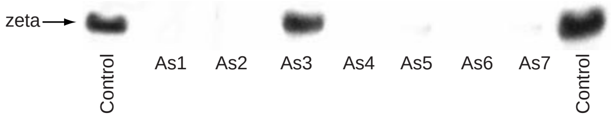

**Figure 1** Representative Western immunoblots showing the expression of CD3 zeta chain in Jurkat cells, following a 4-day incubation with ascitic fluids (diluted 1:4) obtained from ovarian cancer patients. Control lanes correspond to Jurkat cells incubated with medium alone

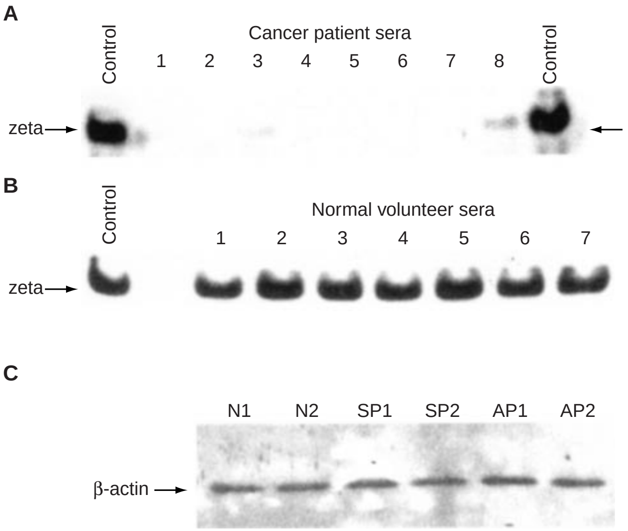

**Figure 2** Representative Western immunoblots indicating the expression of CD3 zeta chain by Jurkat cells, following a 4-day incubation with sera (diluted 1:4) obtained from (**A**) ovarian cancer patients and (**B**) normal female volunteers. Control lanes correspond to Jurkat cells incubated with medium alone. Panel **C** presents the effect of sera from representative normal volunteers (N1, N2), cancer patients' sera (SP1, SP2) and ascites (AP1, AP2) on the expression of β-actin by Jurkat cells

### Statistical analysis

The reactive bands for zeta expression were quantitated by densitometry and the relative absorbances for these bands were standardized to a control lane included within each gel. All relative absorbance determinations were performed at least twice and the mean ± standard deviation for each sample was calculated. Differences between the groups were compared by Student's *t*-test and a two-way ANOVA. For expression of mRNA, the ratio of the signal for the zeta chain to that for β-action was calculated and the zeta/actin ratios obtained for treated Jurkat cells were compared with untreated Jurkat cells.

### RESULTS

### Zeta chain suppression by ovarian cancer-derived ascitic fluid

To define the presence of a factor in the ascitic fluids of women with ovarian cancer, which could modulate TcR-zeta chain levels, the expression of zeta chains was assessed in Jurkat cells, incubated in media supplemented with 25% ascites for 4 days. The expression TcR-zeta was evaluated by Western immunoblot and the representative blots are shown in Figure 1. While suppression of zeta chain expression was observed in all cases (i.e. with ascitic fluids from different patients ($n$ = 15)), it was variable, ranging from 10 to 96% of control zeta levels (72.77 ± 4.98, mean ± SEM).

### Zeta chain suppression by cancer sera vs. normal sera

Since the presence of ascites could diminish the levels of zeta chain, the presence of similar activity in the sera of these patients was also evaluated. The modulation of zeta chain expression was assessed by Western immunoblot in Jurkat cells, incubated in media supplemented with 25% serum for 4 days. Representative blots are presented in Figure 2. Zeta chain expression was suppressed greater than 80% by 27/30 patients' sera tested. In contrast, sera from normal volunteers failed to exhibit significant suppression under the assay conditions. As a control, no significant suppression of β-actin levels were observed in Jurkat cells incubated with patient sera or ascitic fluids.

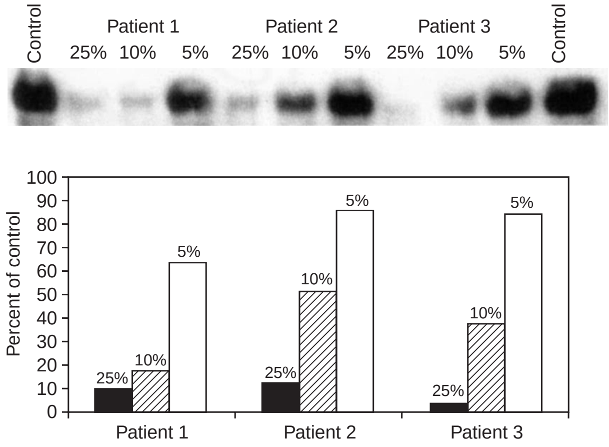

**Figure 3** Representative Western immunoblots indicating the expression of CD3 zeta chain by Jurkat cells, following a 4-day incubation with 3 sera (diluted 1:4, 1:10 and 1:20) obtained from women with ovarian cancer. The control lane corresponds to T-cell incubated with medium alone

*© 2001 Cancer Research Campaign*

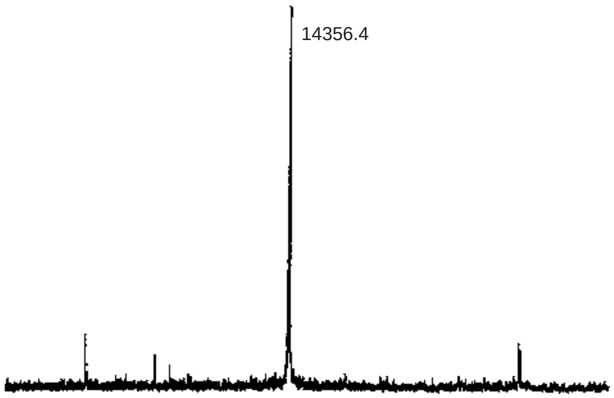

**Figure 4** MADLI-TOF profile obtained from the ascites-derived suppressor of CD3-zeta expression

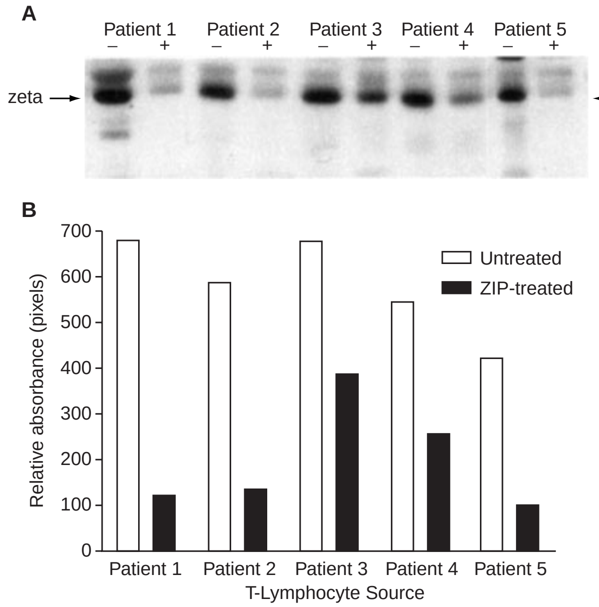

**Figure 5** Panel **A**: Western immunoblots indicating the expression of TcR/CD3 zeta chain by T-lymphocytes obtained from non-tumour bearing female volunteers, following a 4-day incubation in the presence (+) or absence (–) of the ascites-derived zeta inhibitory protein (10 ng $ml^{-1}$). Panel **B**: Level of CD3-zeta expression by these normal T-cells determined by densitometry

### Dose-dependence of zeta suppression

To determine whether suppression of the zeta expression by body fluids obtained from women with ovarian carcinoma was dose-dependent, Jurkat cells were incubated with increasing concentrations of serum or ascitic fluids. As Figure 3 illustrates, the suppression of zeta chain expression was dose-dependent between 5 and 25% of serum added to the culture medium. This observation was consistent for all 12 patients evaluated.

### Characterization of a patient-derived zeta suppression factor

Since the presence and dose-dependence of the zeta chain suppressing activity were observed in most of the patient-derived fluids studied, the next step was directed at purification and characterization of the factor responsible for this activity, in terms of molecular weight, amino acid composition, and sequence. Based on the zeta chain expression bioassay, the partial purifications of the factor from the 5 ascitic fluids yielded $3–4 \times 10^5$-fold purifications following the final RP-HPLC step. Following partial purification, this factor was characterized by mass spectrometry. Determination of the molecular weight of the patient-derived factor by MALDI-TOF yielded a mass of approximately 14KD (Figure 4). Analysis of the amino acid composition of this factor verified the proteinaceous nature of the factor; however, subsequent attempts to sequence this factor by internal sequencing produced only a single sequencable peptide ([K,R]TYELYADXLI), which was not significantly homologous with known proteins based on BlastP analysis.

### Verification of CD3-zeta suppression in normal lymphocytes

The ascites-derived suppressive factors significantly inhibited the expression of CD3-zeta in Jurkat cells; however, while they may express many characteristics of normal T-lymphocytes, these cultured cells are derived from a human T-cell lymphoma and the observed modulation of CD3-zeta might be an artifact of these aberrant cells. The suppression of CD3-zeta chain expression was analysed by Western immunoblot in normal PBLs, incubated with zeta inhibitory protein (10 ng $ml^{-1}$) for 4 days and followed by T-cell isolation (Figure 5). Using pooled, purified inhibitor, the CD3-zeta chain expression of normal T-cells was suppressed by an average of 65%; 581.6 ± 107.2 (mean pixels ± SD) for the untreated T-cells versus 201.2 ± 121.0 for the treated T-cells ($P = 0.0079$). In contrast, an analogous fraction from normal serum failed to exhibit significant suppression under the assay conditions (data not shown).

### Specificity of suppressive effects

The serum-mediated suppression of CD3-zeta could be the consequence of a specific suppression of CD3-zeta (or a limited number of other proteins) or it may represent a non-specific suppression of total protein synthesis within the T-cell. To explore the selectivity of the suppression, expressions of 2 TcR-dependent kinases essential for T-cell activation, *lck* and ZAP-70, were analysed in addition to the zeta chain expression following incubation of Jurkat cells with the partially purified factors from the ascitic fluids of 5 different ovarian cancer patients. The expression level of each signalling molecule was compared to its expression level in Jurkat cells incubated with the analogous fraction obtained from a control

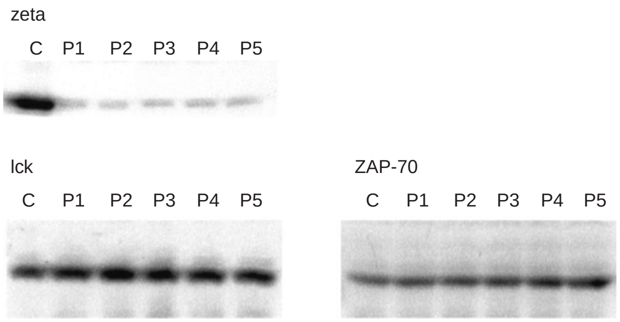

**Figure 6** Western immunoblots indicating the expression of zeta chain (Panel **A**), lck (Panel **B**) and ZAP-70 (Panel **C**) by Jurkat cells, following a 4-day incubation with purified ZIP (10 ng $ml^{-1}$), obtained from 5 women with ovarian cancer. The control lane corresponds to T-cell incubated with the analogous fraction from control serum

© 2001 Cancer Research Campaign

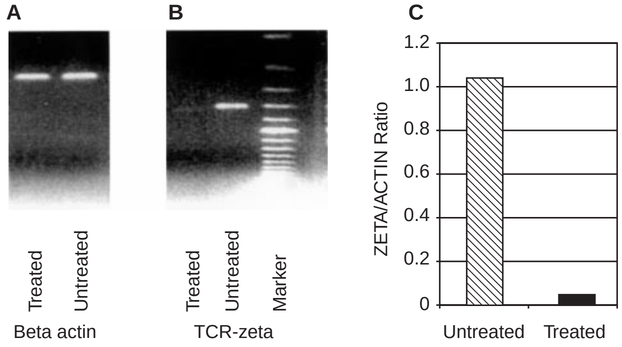

**Figure 7** Expression of mRNA for the CD3 zeta chain and β-action in Jurkat cells, following a 4-day incubation with the partially purified ascites-derived suppressor factor (10 ng $ml^{-1}$). The conrol lane corresponds to T-cell incubated with the analogous fraction from control serum serum. Representative data comparing the effects of the factor on these proteins are presented in Figure 6. Neither *lck* nor ZAP-70 was suppressed, indicating the effects of zeta inhibitory factor are not the result of a ubiquitous inhibition of total T-cell proteins or of an indiscriminate inhibition of proteins within the T-cell activation pathway.

### Defining the molecular target of ZIP

The loss of CD3-zeta chain observed in cancer can result from either increased degradation of the zeta protein or reduced synthesis of this protein. In our initial study to define the site of zeta chain suppression, the level of CD3-zeta chain mRNA was analysed by RT-PCR. The level of specific mRNA was analysed by 25 cycles of PCR amplification, with β-actin levels used as a control (Figure 7). T-cells treated with the cancer patient-derived material exhibited a suppression of mRNA paralleling the loss of zeta chain protein.

### DISCUSSION

In women with ovarian cancer, suppression of components of the immune system frequently occurs and it might facilitate tumour development. Patients with advanced malignancies exhibit progressively impaired immune responses, indicating that tumour cells have developed mechanisms to subvert the immune system and suppress immune surveillance. Previous studies have demonstrated that alterations in expression and function of the TcR-associated signal transducing zeta-chain are responsible for deficient immune responsiveness of T cells in several types of malignancies, including ovarian and cervical cancers (Lai et al, 1996; Rabinowich et al, 1996; deGruijl et al, 1999). The decreased expression of zeta chain in cancer patients' T lymphocytes has also been observed to correlate with the extent of disease and short survival (Shores et al, 1997). While the loss of zeta chain is a common observation, the mechanisms responsible for the cancer-associated decreased expression of T-cell zeta chain remain unknown.

The zeta chain is responsible for transducing the activation signal from the antigen binding T-cell receptor to the interior of the lymphocyte. It has been suggested that the phosphorylation patterns of the multiple immunoreceptor tyrosine-activation motifs (ITAMs) of the zeta chains determine the selection of specific T-cells during positive and negative selection in the thymus (Hermans and Malissen, 1993; Irving et al, 1993). Thus, the zeta chain appears to play a critical role in the development of the TcR repertoire (Chan et al, 1994). In mature lymphocytes, the absence of zeta chain alters the expression and functions of CD3/TcR complex. T-cells with suppressed zeta chain have been demonstrated to exhibit diminished proliferation and production of cytokines. Decreased zeta chain expression occurs in other disease states, including chronic infections and systemic lupus erythematosus, where deficient zeta chain expression has been proposed to aberrantly modulate T-cell functions (Liossis et al, 1998).

In this study, we demonstrated that sera and ascitic fluids of women with advanced ovarian cancer exhibited the ability to specifically suppress the expression to T-cell CD3-zeta chain (Figures 1 and 2). Similar suppressive activity could not be demonstrated in the sera of non-tumour-bearing female controls. This suppression appeared to decrease zeta chain levels in a dose-dependent fashion (Figure 3), suggesting that this effect may be receptor-mediated. These results suggest that the presence of the zeta inhibitor is associated with the presence of cancer. Recent preliminary studies have indicated that this factor may be tumour-derived, since a similar suppressive factor, in terms of activity and molecular weight, can be demonstrated in conditioned media from cultured ovarian tumour cells, but not in media from normal fibroblasts.

The factor mediating the loss of zeta chain was purified from the ascites fluids of ovarian cancer patients. This zeta inhibitory protein (ZIP) has a molecular weight of approximately 14 kDa, based on mass spectrometry (Figure 4). The suppression of CD3-zeta mediated by ZIP is not an artifact of the use of Jurkat cells, since a similar loss of TcR/CD3-zeta was observed in normal T-cell exposed to purified ZIP.

The patient-associated suppression of T-cell CD3-zeta appeared to be the consequence of a specific suppression or loss of zeta chain expression and not a non-specific suppression of total protein synthesis within the T-cell, since the TcR-associated kinases, lck and ZAP-70, were unaffected by exposure to ZIP (Figure 6), while zeta chain was almost completely suppressed. This selective suppression of TcR/CD3-zeta appears to be induced at the level of the zeta mRNA, since T-cells treated with the cancer patient-derived material exhibited a suppression of mRNA paralleling the loss of zeta chain protein (Figure 7). This finding seems to contradict previous results suggesting that the loss of TcR/CD3-zeta was due to increased degradation and not decreased synthesis (Reichert et al, 1998). It should be noted that this previous conclusion was drawn from in vivo derived lymphocytes that have been exposed to multiple factors within the host, including tumour-derived proteolytic enzymes and factors capable of inducing degradative pathways.

In all of these previous studies addressing tumour-mediated suppression of TcR/CD3-zeta chain, T-lymphocytes have not been exposed to a single, purified factor, whether in vivo with exposure to the tumour-microenvironment or in vitro with exposure to whole tumour cells. In our initial studies using unfractionated ascitic fluids, suppression of both CD3-zeta and ZAP-70 and induction of apoptosis were observed; however, the purified zeta-inhibitory factor failed to suppress ZAP-70 levels or induce cell death. Thus, while multiple pathways can lead to suppression of CD3-zeta either directly or indirectly, it is difficult to define the contribution of each using unfractionated materials.

 © 2001 Cancer Research Campaign

This study has identified a tumour-associated zeta chain-inhibitory factor. However, other potential zeta-modulating factors have been described. In vitro studies on decreased zeta chain expression have demonstrated a role of surface-associated FasL; however, several investigators have questioned a pathway by which cell-associated FasL could induce the systemic loss of CD3-zeta chains observed in ovarian cancer patients. Our preliminary findings have demonstrated circulating FasL-associated with shed tumour membrane vesicles. Similar FasL-containing vesicles have been shown to be shed by overactivated T-lymphocytes and, in contrast to soluble FasL, the particulate FasL retains its ability to mediate activation-induced cell death. Since these vesicles are greater than 50 million Daltons, they would have been removed during our presently described purification. In addition, Maccalli et al (1999) previously demonstrated the modulation of zeta expression by soluble HLA class I molecules derived from melanoma cells. The presence of multiple pathways capable of suppressing the level of TcR/CD3-zeta chains might explain the apparent less pronounced inhibition of zeta levels with the purified protein than with the unfractionated ascites.

The characteristics of these proteins and the mechanism and specificity of zeta suppression demonstrated in this report suggest that these factors are distinct. By defining the mechanism through which our protein modulates TcR/CD3-zeta chain levels, may ultimately provide a target for the prevention of the suppressive influences of the tumour microenvironment observed in ovarian cancer. By preventing T-cell zeta chain loss, effective anti-tumor cellular immune responses can be generated and protective immune responses restored. Thus, understanding the molecular mechanisms of tumour-mediated T-cell dysfunction is likely to have impact on future developments in the prevention, diagnosis and therapy of cancer.

### REFERENCES

Bast RC and Knapp RC (1984) Immunologic approaches to the management of ovarian carcinoma. *Sem Oncol* **11**: 264–274

Braun DP and Harris JE (1983) Serial immune function testing to predict clinical disease relapse in patients with solid tumors. *Cancer Immunol Immunoth* **15**: 165–171

Brooks B and Rees RC (1988) Human recombinant IL-4 suppresses the induction of human IL-2 induced lymphocyte activated killer activity. *Clin Exp Immunol* **74**: 162–165

Brown R, Clugston C, Burns P, Edlin A, Vasey P, Vojtesek B and Kaye SB (1993) Increased accumulation of p53 protein in cisplatin resistant ovarian cell lines. *Int J Cancer* **55**: 678–684

Chan AC, Desai DM and Weiss A (1994) The role of protein tyrosine kinases and protein tyrosine phosphatases in T cell antigen receptor signal transduction. *Ann Rev Immunol* **12**: 555–592

deGruijl TD, Bontkes HJ, Peccatori F, Gallee MPW, Helmerhorst TJM, Verheijen RHM, Aarbiou J, Mulder WMC, Walboomers JMM, Meijer CJLM, van de Vange N and Scheper RJ (1999) Expression of CD3-ζ on T-cells in primary cervical carcinoma and metastasis-positive and negative pelvic lymph nodes. *Brit J Cancer* **79**: 1127–1132

Hellstrom KE and Hellstrom I (1991) Principles of tumor immunity: Tumor antigens. In: *Biologic therapy of cancer*, DeVita VT, Hellman S and Rosenberg SA (eds), pp 35–52. Lippincott: New York

Hermans MHA and Malissen B (1993) The cytoplasmic tail of the T cell receptor ζ chain is dispensable for antigen-mediated T cell activation. *Eur J Immunol* **9**: 2757–2762.

Irving BA, Chan AC and Weiss A (1993) Functional characteristics of a signal transducing motif present in the T cell antigen receptor ζ chain. *J Exp Med* **177**: 1093–1103

Kersh EN, Kersh GJ and Allen PM (1999) Partially phosphorylated T cell receptor ζ molecules can inhibit T cell activation. *J Exp Med* **190**: 1627–1636

Kuss I, Saito T, Johnson JT and Whiteside TL (1999) Clinical significance of decreased z chain expression in peripheral blood lymphocytes of patients with head and neck cancer. *Clin Cancer Res* **5**: 329–334

Lai P, Rabinowich H, Crowley-Nowick, PA, Bell MC, Mantovani G and Whiteside TL (1996) Alterations in expression and function of signal-transducing proteins in tumor-associated T and natural killer cells in patients with ovarian cancer. *Clin Cancer Res* **2**: 161–173

Laemmli UK (1970) Cleavage of structural proteins during the assembly of the head of the bacteriophage T4. *Nature* **227**: 680–685

Liossis SC, Ding XC, Xuan Z, Dennis GJ and Tsokos GC (1998) Altered pattern of TCR/CD3-mediated protein-tyrosyl phosphorylation in T cells from patients with systemic lupus erythematosus: Deficient expression of the T cell receptor zeta chain. *J Clin Invest* **101**: 1448–1457

Maccalli C, Pisarra P, Wegetti C, Sensi M, Parmiani G and Anichini A (1999) Differential loss of T cell signaling molecules in metastatic melanoma patients' T lymphocyte subsets expressing distinct TCR variable regions. *J Immunol* **163**: 6912–6923

Mulder WMC, Bloemena E, Stukart MJ, Kummer JA, Wagstaff J and Scheper RJ (1997) T-cell receptor zeta and granzyme B expression in mononuclear cell infiltrates in normal colon mucosa and colon carcinoma. *Gut* **40**: 113–119

Old LJ (1981) Cancer immunology: The search for specificity-G. H. A. Clowes Memorial lecture. *Cancer Res* **41**: 361–375

Rabinowich H, Suminami Y, Reichert TE, Crowley-Nowick P, Bell M, Edwards R and Whiteside TL (1996) Expression of cytokine genes or proteins and signaling molecules in lymphocytes associated with human ovarian carcinoma. *Int J Cancer* **68**: 276–284

Rabinowich H, Reichert TE, Kashii Y, Gaastman BR, Bell MC and Whiteside TL (1998) Lymphocyte apoptosis induced by Fas ligand-expressing ovarian carcinoma cells: implicates for altered expression of T cell receptor in tumor associated lymphocytes. *J Clin Invest* **101**: 2579–2588

Reichert TE, Rabinowich H, Johnson JT and Whiteside TL (1998) Immune cells in the tumor microenvironment: Mechanisms responsible for signaling and functional defects. *J Immunother* **21**: 295–306

Shores EW, Tran T, Grinberg A, Sommers CL, Shen H and Love PE (1997) Role of the multiple T cell receptor (TCR)-ζ chain signaling motifs in selection of the T cell repertoire. *J Exp Med* **185**: 893–900.

Suminami Y, Elder EM, Lotze MT and Whiteside TL (1995) In situ interleukin-4 gene expression in cancer patients treated with genetically modified tumor vaccine. *J Immunother* **17**: 238–248

Tartour E, Latour S, Mathiot C, Thiounn N, Mosseri V, Joyeux I, D'Enghien CD, Lee R, Debre B and Fridman WH (1995). Variable expression of CD3-ζ chain in tumor-infiltrating lymphocytes derived from renal cell carcinoma: Relationship with TIL phenotype and function. *Int J Cancer* **63**: 205–212

Yasumura S, Amoscato A, Hirabayashi H, Lin WC and Whiteside TL (1994) Proliferation of hematopoietic cell lines induced by a soluble factor derived from human squamous cell carcinomas of the head and neck. *Cancer Immunol Immunother* **39**: 407–415

Zea AH, Cutri BD, Longo DL, Alvord WG, Strobl SL, Mizoguchi H, Creekmore SP, O'Shea JJ, Powers GC, Urba WJ and Ochoa AC (1995) Alterations in T cell receptor and signal transduction molecules in melanoma patients. *Clin Cancer Res* **1**: 1327–1335

*© 2001 Cancer Research Campaign*
